# The Protective Effect of MicroRNA-320 on Left Ventricular Remodeling after Myocardial Ischemia-Reperfusion Injury in the Rat Model

**DOI:** 10.3390/ijms151017442

**Published:** 2014-09-29

**Authors:** Chun-Li Song, Bin Liu, Hong-Ying Diao, Yong-Feng Shi, Yang-Xue Li, Ji-Chang Zhang, Yang Lu, Guan Wang, Jia Liu, Yun-Peng Yu, Zi-Yuan Guo, Jin-Peng Wang, Zhuo Zhao, Jian-Gen Liu, Yi-Hang Liu, Zhi-Xian Liu, Dan Cai, Qian Li

**Affiliations:** Department of Cardiology, the Second Hospital of Jilin University, Ziqiang Street No. 218, Nanguan District, Changchun 130000, China; E-Mails: liubinlb220@163.com (B.L.); diaohongying220@163.com (H.-Y.D); shiyongfeng1017@126.com (Y.-F.S.); yangxueli628@163.com (Y.-X.L.); zhangjichang220@163.com (J.-C.Z.); luyangly220@163.com (Y.L.); wangguan628@163.com (G.W.); jialiu_joe@126.com (J.L.); yuyunpeng013@126.com (Y.-P.Y.); guoziyuan628@163.com (Z.-Y.G.); wangjinpeng628@163.com (J.-P.W.); zhaozhuo628zz@163.com (Z.Z.); liujiangen220@163.com (J.-G.L.); liuyihang628@163.com (Y.-H.L.); liuzhixian928@163.com (Z.-X.L.); caidancd220@163.com (D.C.); liqianlq220@163.com (Q.L.)

**Keywords:** microRNA-320, myocardial ischemia-reperfusion injury, left ventricular remodeling

## Abstract

The primary objective of this study investigated the role of microRNA-320 (miR-320) on left ventricular remodeling in the rat model of myocardial ischemia-reperfusion (I/R) injury, and we intended to explore the myocardial mechanism of miR-320-mediated myocardium protection. We collected 120 male Wistar rats (240–280 g) in this study and then randomly divided them into three groups: (1) sham surgery group (sham group: *n* = 40); (2) ischemia-reperfusion model group (I/R group: *n* = 40); and (3) I/R model with antagomir-320 group (I/R + antagomir-320 group: *n* = 40). Value changes of heart function in transesophageal echocardiography were recorded at various time points (day 1, day 3, day 7, day 15 and day 30) after surgery in each group. Myocardial sections were stained with hematoxylin and eosin (H&E) and examined with optical microscope. The degree of myocardial fibrosis was assessed by Sirius Red staining. Terminal dUTP nick end-labeling (TUNEL) and qRT-PCR methods were used to measure the apoptosis rate and to determine the miR-320 expression levels in myocardial tissues. Transesophageal echocardiography showed that the values of left ventricular ejection fraction (LVEF), left ventricular fractional shortening (LVFS), left ventricular systolic pressure (LVSP) and ±d*p*/d*t*_max_ in the I/R group were obviously lower than those in the sham group, while the left ventricular end-diastolic pressure (LVEDP) value was higher than that in the sham group. The values of LVEF, LVFS, LVSP and ±d*p*/d*t*_max_ showed a gradual decrease in the I/R group, while the LVEDP value showed an up tendency along with the extension of reperfusion time. The H&E staining revealed that rat myocardial tissue in the I/R group presented extensive myocardial damage; for the I/R + antagomir-320 group, however, the degree of damage in myocardial cells was obviously better than that of the I/R group. The Sirius Red staining results showed that the degree of myocardial fibrosis in the I/R group was more severe along with the extension of the time of reperfusion. For the I/R + antagomir-320 group, the degree of myocardial fibrosis was less severe than that in the I/R group. Tissues samples in both the sham and I/R + antagomir-320 groups showed a lower apoptosis rate compared to I/R group. The qRT-PCR results indicated that miR-320 expression in the I/R group was significantly higher than that in both the sham and I/R + antagomir-320 groups. The expression level of miR-320 is significantly up-regulated in the rat model of myocardial I/R injury, and it may be implicated in the prevention of myocardial I/R injury-triggered left ventricular remodeling.

## 1. Introduction

Cardiovascular disease is the leading cause of death in both the United States and among worldwide populations, according to the World Health Organization [1]. Ischemic heart disease (IHD) is classified as the leading cause of global mortality by the Global Burden of Disease study from various types of cardiovascular diseases [2]. IHD is the leading cause of death and disability worldwide, with mortality rates of IHD ranging from 13 per 100,000 in Kiribati to 456 per 100,000 in Turkmenistan, and the IHD burden is higher in the Middle East, North America, Australia and much of Europe, causing great health, economic and personal burdens [3]. Reperfusion of the occluded artery was applied to the current treatment of ICH, but reperfusion may induce myocardial ischemia-reperfusion (I/R) injury, including arrhythmias, myocardial stunning, microvascular obstruction and cardiomyocyte death [3,4]. Additionally, I/R injury may be involved in the development of left ventricular remodeling associated with cardiac myocyte death, vascular rarefaction, fibrosis, inflammation and electrophysiological remodeling, resulting in the pathophysiology of advancing heart failure and sudden cardiac death [5,6,7,8]. Recent investigations found molecular and cellular mechanisms and suggested that microRNAs (miRs) may be crucial in the regulation of I/R injury-induced cardiac injury and dysfunction, governing the development of ventricular remodeling after I/R injury [9,10,11].

Numerous evidence suggested that miRs showed evidence of significant roles in regulating gene expression, which are implicated in the cardiac development and pathological process of cardiovascular diseases [9,12,13]. The miRs had functional roles in regulating the response to I/R injury by altering the expression of various key elements in cell survival and apoptosis; thus, miRs could be potential therapeutic targets for the treatment of heart disease [12,13,14]. miRNAs are differentially expressed in the failing myocardium and may govern diverse functions in cardiac remodeling process by targeting genes and signaling elements [15,16,17,18,19]. More specifically, miR-320, a member of the miR family, plays functional roles in the inhibition of cell division, acting in stromal fibroblasts and regulating glycolysis, which may play important roles in the fibrosis development associated with left ventricular remodeling and in the energy metabolism of IHD [20,21,22]. Subsequently, miR-320 expression is up-regulated in patients with failing hearts in contrast to the healthy controls and is associated with potential cardiovascular target genes [23]. It has been revealed that miR-320 expression in human left ventricular samples was significantly altered in the heart disease group and the control group, showing evidence of the over-expression of miR-320 in ischemic cardiomyopathy and aortic stenosis [24]. Over-expression of miR-320 enhances cell death and apoptosis in cultured adult rat cardiomyocytes on simulated I/R injury; thus, down-regulation of miR-320 expression may be involved in the protection against cardiac hypertrophic remodeling, but the molecular mechanisms are still not completely understood [25]. In this study, we established a rat model of I/R injury to evaluate the potential role of miR-320 in the protection of left ventricular remodeling resulting from myocardial injury, to find antisense strategies or pharmacological approaches for conferring protection against I/R injury; thus, we expanded our understanding of the cellular function and pathophysiological roles of miR-320.

## 2. Results

### 2.1. Differences in Cardiac Function of Three Groups

Transesophageal echocardiography was applied to detect cardiac function in the sham group, the I/R group and the I/R + antagomir-320 group based on the LVEF, LVFS, LVSP, LVEDP and ±d*p*/d*t*_max_ in each group at different time points. The result has shown no significant differences of LVEF, LVFS, LVSP, LVEDP and ±d*p*/d*t*_max_ at different time points in the sham group. Compared with the shame group, the values of LVEF, LVFS, LVSP and ±d*p*/d*t*_max_ in the I/R group at different time points were obviously lower, while the LVEDP value was higher (all *p* < 0.05). Furthermore, along with the extension of reperfusion time, the values of LVEF, LVFS, LVSP and ±d*p*/d*t*_max_ in the I/R group gradually declined, but the LVEDP value gradually increased. Nevertheless, the values of LVEF, LVFS, LVSP and ±d*p*/d*t*_max_ of the I/R + antagomir-320 group were higher than those of the I/R group at each time point, but the value of LVEDP was lower than that of the I/R group at each time point (all *p* < 0.05) (Table 1).

**Table 1:** Differences in cardiac function of three groups. LVEF, left ventricular ejection fraction; LVFS, left ventricular fractional shortening; LVSP, left ventricular systolic pressure.

**Time Point**		**LVEF (%)**						**LVFS (%)**						**LVSP (mmHg)**				
		**1**		**2**		**3**		**1**		**2**		**3**		**1**		**2**		**3**
day 1		83.5 ± 2.7		72.2 ± 2.4 *		80.6 ± 2.9 ^#^		47.3 ± 4.2		36.3 ± 1.9 *		42.6 ± 3.1 ^#^		128 ± 4		111 ± 8 *		119 ± 7 ^#^
day 3		84.3 ± 2.7		63.2 ± 1.0 *		77.2 ± 2.2 ^#^		47.9 ± 4.2		30.1 ± 1.2 *		40.4 ± 3.3 ^#^		130 ± 8		100 ± 8 *		111 ± 7 ^#^
day 7		85.3 ± 2.6		56.7 ± 1.1 *		70.7 ± 2.5 ^#^		48.3 ± 4.5		25.5 ± 1.3 *		35.26 ± 2.2 ^#^		131 ± 8		96 ± 8 *		104 ± 7 ^#^
day 15		85.2 ± 2.5		52.6 ± 1.3 *		64.7 ± 2.2 ^#^		48.2 ± 3.9		23.4 ± 1.0 *		30.8 ± 2.1 ^#^		129 ± 7		92 ± 5 *		100 ± 6 ^#^
day 30		84.7 ± 2.7		46.9 ± 1.1 *		59.3 ± 2.1 ^#^		48.4 ± 3.5		21.2 ± 1.4 *		29.7 ± 1.9 ^#^		128 ± 6		88 ± 7 *		97 ± 6 ^#^
**Time Point**	**LVEDP (mmHg)**						**+d*p*/d*t*_max_ (mmHg/s)**						**−d*p*/d*t*_max_ (mmHg/s)**					
	**1**		**2**		**3**		**1**		**2**		**3**		**1**		**2**		**3**	
day 1	4.5 ± 0.8		6.5 ± 1.1 *		5.3 ± 0.9 ^#^		12,003 ± 789		9258 ± 753 *		11,113 ± 719 ^#^		8367 ± 693		7442 ± 653 *		8004 ± 598 ^#^	
day 3	4.6 ± 0.6		6.8 ± 0.7 *		5.9 ± 0.9 ^#^		11,936 ± 823		8843 ± 672 *		9656 ± 679 ^#^		8438 ± 703		6982 ± 637 *		7689 ± 655 ^#^	
day 7	4.3 ± 0.6		7.1 ± 0.7 *		6.8 ± 0.8 ^#^		12,538 ± 815		8121 ± 648 *		9100 ± 772 ^#^		8257 ± 654		6478 ± 623 *		7339 ± 641 ^#^	
day 15	4.3 ± 0.7		7.8 ± 1.1 *		7.1 ± 0.8 ^#^		12,784 ± 891		7934 ± 711 *		8807 ± 707 ^#^		8354 ± 697		6002 ± 605 *		6891 ± 646 ^#^	
day 30	4.5 ± 0.7		8.9 ± 1.3 *		7.9 ± 0.9 ^#^		12,034 ± 901		7453 ± 618 *		7887 ± 710 ^#^		8369 ± 598		5840 ± 579 *		6331 ± 622 ^#^	

1, the sham group; 2, the ischemia-reperfusion (I/R) group; 3, the I/R + antagomir-320 group; * *p* < 0.05 compared with the sham group; ^#^ *p* < 0.05 compared with the I/R group.

### 2.2. Degree of Damage in Myocardial Cells

**Figure 1:**
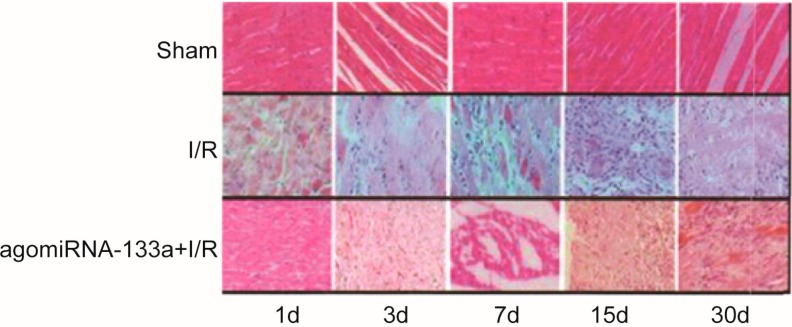
Myocardial cells of three groups at various time points.

The H&E staining revealed that the rat myocardial tissue of the sham group had no obvious myocardial injury. Normal myocardial cell and nuclei, mild interstitial congestion and a few wavy fibers were observed in the sham group without significant differences at different time points. Compared with the sham group, the I/R group exhibited extensive myocardial damage—disruption and lysis of the myocardial cell, proliferation of fiber cells, formation of an infarct scar region—while the degree of damage in myocardial cells of the I/R + antagomir-320 group was significantly better than that of the I/R group (Figure 1).

### 2.3. Evaluation of Myocardial Fibrosis of Three Groups

**Figure 2:**
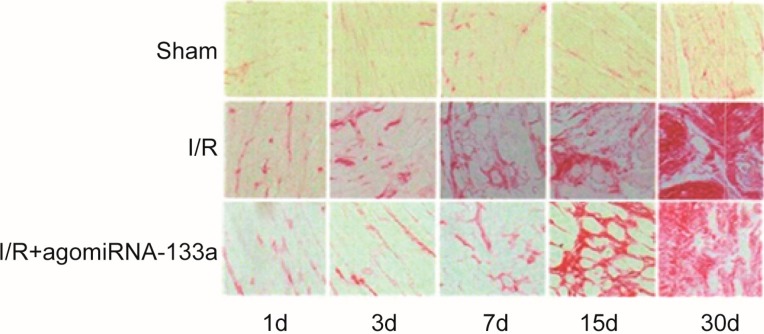
Degree of myocardial fibrosis of three groups at various time points.

Only mild myocardial fibrosis was observed in the sham group with no obvious differences at different time points (all *p* > 0.05). Compared with the sham group, the degree of myocardial fibrosis in the I/R group was more severe along with the extension of reperfusion time, especially from day 3 to day 30 (day 1: I/R *vs.* sham: (5.60 ± 2.30) (%) *vs.* (1.64 ± 0.41) (%), *p* < 0.05; day 30: I/R *vs.* sham: (29.81 ± 6.86) (%) *vs.* (1.74 ± 0.35) (%), *p* < 0.001). However, the degree of myocardial fibrosis in the I/R + antagomir-320 group was less severe than that in the I/R group at each time point (all *p* < 0.05) (Figure 2 and Table 2).

**Table 2:** Evaluation of the degree of myocardial fibrosis in three groups.

Group	Degree of Myocardial Fibrosis (%)				
	Day 1	Day 3	Day 7	Day 15	Day 30
Sham	1.83 ± 0.47	1.64 ± 0.41	1.57 ± 0.37	1.64 ± 0.38	1.74 ± 0.35
I/R	2.03 ± 0.81	5.60 ± 2.30 *	28.54 ± 7.12 *	36.21 ± 7.50	37.54 ± 5.71
I/R + antagomir-320	1.96 ± 0.66	4.68 ± 1.55 ^#^	19.42 ± 6.11 ^#^	25.10 ± 8.30	29.81 ± 6.86

* *p* < 0.05 compared with the sham group; ^#^ *p* < 0.05 compared with the I/R group.

### 2.4. Detection of Apoptosis by Terminal dUTP Nick End-Labeling (TUNEL) Staining

**Figure 3:**
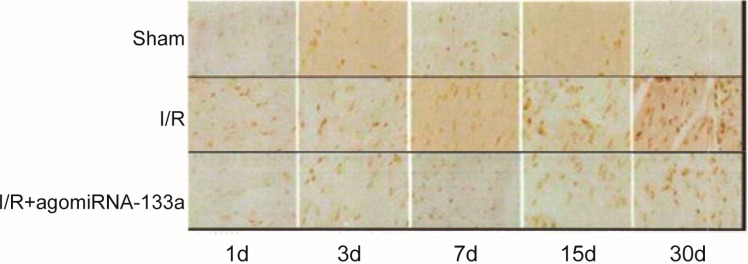
Detection of the apoptosis rate of myocardial cells by TUNEL staining.

TUNEL staining was applied to detect the apoptosis of myocardial cells in the three groups. No obvious myocardial cells were found in the sham group at different time points. There was a large amount of apoptosis myocardial cells observed in the I/R group compared to the sham group, especially at the day 3 after reperfusion (I/R *vs.* sham: (54.6 ± 7.3) (%) *vs.* (5.2 ± 2.1) (%), *p* < 0.05). The large amount of apoptosis may be due to the ischemia reperfusion injury, which was in line with the result of H&E staining. Compared with the I/R group, the apoptosis rate was significantly decreased in the I/R + antagomir-320 group (all *p* < 0.05) (Figure 3 and Table 3).

**Table 3:** The apoptosis rate of myocardial cells in three groups by terminal dUTP nick end-labeling (TUNEL) staining.

Group	Apoptosis Rate (%)				
	Day 1	Day 3	Day 7	Day 15	Day 30
Sham	5.6 ± 2.7	4.8 ± 2.4	5.4 ± 1.8	6.3 ± 2.2	6.4 ± 1.6
I/R	32.6 ± 5.4 *	54.6 ± 7.3 *	42.7 ± 6.8 *	38.2 ± 7.5 *	33.4 ± 5.3 *
I/R + antagomir-320	18.7 ± 3.3 ^#^	30.5 ± 6.4 ^#^	33.3 ± 8.7 ^#^	29.5 ± 8.7 ^#^	22.4 ± 4.8 ^#^

* *p* < 0.05 compared with the sham group; ^#^ *p* < 0.05 compared with the I/R group.

### 2.5. miR-320 Expression in Myocardial Tissue

qRT-PCR was applied to detect the expression of miR-320 in the three groups. Table 4 demonstrated significantly higher expression of miR-320 in the I/R group than that of the sham group at each time point (all *p* < 0.05), especially at 3 d after surgery (0.540 ± 0.134). Meanwhile, we observed that the expression of miR-320 dramatically decreased in the I/R + antagomir-320 group compared with the I/R group during day 3 to day 30 after surgery (all *p* < 0.05).

**Table 4:** Expression of miR-320 in the myocardial tissue of three groups at five time points.

Group	miR-320 (2^−∆∆*C*t^)				
	Day 1	Day 3	Day 7	Day 15	Day 30
Sham	1.00 ± 0.00	1.00 ± 0.00	1.00 ± 0.00	1.00 ± 0.00	1.00 ± 0.00
I/R	0.60 ± 0.13 *	0.52 ± 0.13 *	0.63 ± 0.10 *	0.76 ± 0.09 *	0.72 ± 0.10 *
I/R + agomiR-133a	0.86 ± 0.33	1.55 ± 0.14 ^#^	1.73 ± 0.27 ^#^	1.63 ± 0.11 ^#^	1.91 ± 0.95 ^#^

* *p* < 0.05 compared with the sham group; ^#^ *p* < 0.05 compared with the I/R group.

## 3. Discussion

Ischemic heart disease has been widely accepted as one of the most common and detrimental diseases, and surgical treatments for patients with ischemic heart disease do not mean complete myocardial ischemic reperfusion (I/R) and a normal internal environment [25]. It has also been reported that severe myocardial I/R injury-induced ventricular remodeling may occur after myocardial I/R injury [26]. The possible mechanism may be that myocardial cell death after myocardial I/R injury leads to myocardial remodeling [27]. Therefore, it is very important to find a method to improve the myocardial I/R injury-induced ventricular remodeling for the treatment of ischemic heart disease. Furthermore, myocardial microvascularity has been the basis of myocardial microcirculatory, and the dysfunction of myocardial microvascularity or the abnormality of related regulators may affect the function of myocardial microcirculation and have effects on myocardial perfusion [28,29]. Microcirculatory disturbance caused by coronary ischemia may induce myocardial necrosis [30]. On the other hand, the disruption of myocardial microcirculation or angiogenesis may serve as a potential risk factor in the pathogenesis of cardiomyopathy or ischemic heart diseases [31,32,33]. In this regard, angiogenesis or disruption of myocardial microcirculation are important characteristics in the pathophysiology of heart diseases. Altered expression of certain genes may result in the changes of regulatory protein in cells, which could be the basis of the pathology of ventricular remodeling that is triggered by myocardial I/R injury; so, the detection of the gene level may provide new ideas for the clinical treatment of ventricular remodeling [34,35]. A variety of miRs negatively regulate gene expression and may reduce the risk of myocardial IRI and ventricular remodeling [9,36]. The purpose of this study is to investigate the role of miR-320 in myocardial I/R injury and to observe miR-320 expression in left ventricular remodeling after myocardial I/R injury. We also explored whether miR-320 is implicated in the process to preserve cardiac function.

In this study, our transesophageal echocardiography results have observed that the values of LVEF, LVFS, LVSP and ±d*p*/d*t*_max_ in the I/R group were apparently lower than those in the sham group, while the value of LVEDP was higher than that in the sham group. Furthermore, the values of LVEF, LVFS, LVSP and ±d*p*/d*t*_max_ in the I/R group gradually declined, but the LVEDP value gradually increased along with the extension of the time of reperfusion. Nevertheless, the values of LVEF, LVFS, LVSP and ±d*p*/d*t*_max_ in the I/R + antagomir-320 group were significantly higher when compared to the I/R group, indicating that the treatment of miR-320 may improve the heart function after myocardial I/R injury. Ischemic heart disease patients after therapy often suffer from myocardial reperfusion, which may cause acute myocardial damage and ventricular remodeling [4]. Reoxygenation and the restoration of blood flow were frequently connected with an aggravation of tissue injury, that is to say, ischemia reperfusion injury [9]. Moreover, myocardial I/R injury may lead to cyto-architectonic alterations and affect the cellular functions, and this was closely related to the myocardial cell apoptosis and myocardial necrosis [26,37]. In addition, hemodynamic changes are correlated with the development of arrhythmias [38]. Arrhythmogenesis is attributed to enhanced trigger activity, which could result from the alterations in different ion channels, especially the peculiar instability in Ca^2+^ handling [39]. miRs function significantly in the regulation of several properties of cardiac physiology and excitability and Ca^2+^ handling, and various studies have unveiled the essential role of miRs in modulating cardiac excitability and arrhythmogenesis [40,41]. Numerous studies have suggested that myocardial I/R injury may induce the alterations of cardiac hemodynamic parameters, which may affect heart function [42,43]. Fan *et al.* observed that the values of LVEF and LVFS were decreased in the myocardial I/R injury tissues when compared with the sham controls, which was in line with our study results [44]. Furthermore, Qin and his colleagues have demonstrated that miR-21 can apparently improve the values of cardiac hemodynamic parameters and suggested that miR-21 may improve the heart function in mice with I/R injury [17]. It has been reported that circulating cardio-enriched miRs might be negatively associated with LVEF [45]; hence, we suspected that miR-320 expression may also regulate the changes of cardiac hemodynamics. On the other hand, an increased miR-320 expression level has been reported to promote cell apoptosis and to inhibit neoangiogenesis, which may be related to myocardial I/R injury [46]. Therefore, myocardial I/R injury may finally lead to the impairment of heart function, which was in agreement with our transesophageal echocardiography results.

In addition, our H&E staining results revealed that the I/R group exhibited extensive myocardial damage when compared with the sham group, while the degree of damage in the myocardial cells of the I/R + antagomir-320 group was significantly better than that of the I/R group. These results suggested that the decreased miR-320 expression level may decrease the degree of myocardial damage, reduce the risk of ventricular remodeling after surgery and, then, improve heart function. The reason why miR-320 was associated with the degree of myocardial damage may be that over-expression of miR-320 may downregulate the heat-shock protein 20 (Hsp20), which may protect the heart against myocardial I/R injury, while the decreased expression of miR-320 may reduce infarct size [15,22]. Another primary result of this study demonstrated that the degree of myocardial fibrosis in the I/R group was more severe than that in sham group. Furthermore, we found that myocardial fibrosis got worse along with the extension of the time of reperfusion, especially from day 3 to day 30, whereas the degree of myocardial fibrosis in the I/R + antagomir-320 group was less severe than that in the I/R group at each time point. These findings illustrated that myocardial I/R injury may result in the proliferation of granulation tissues and even result in fibroblastic proliferation. It has been widely reported that myocardial fibrosis may be a risk factor for cardiac insufficiency, which may have a negative influence on heart function [47]. Fibrosis remodeling occurred after myocardial I/R injury, which may induce ventricular remodeling and myocardial fibrosis and, finally, may lead to ventricular configuration alterations and diastolic functional impairment [48,49]. In addition, we found that the degree of fibrosis remodeling in the I/R + antagomir-320 group was apparently lower than that in the I/R group at each time point, implying that the downregulation of the expression of miR-320 may inhibit the formation of myocardial fibrosis after myocardial I/R.

Our TUNEL results also indicated that the apoptosis rate of myocardial cells in the I/R group was significantly higher than that in sham group, while the rate of myocardial cell apoptosis in the I/R + antagomir-320 group was apparently lower than that in the I/R group. These outcomes have suggesting that myocardial I/R may induce myocardial cell apoptosis and that treatment with miR-320 can reduce myocardial cell apoptosis. An increased expression level of miR-320 may lead to cell apoptosis or death and may increase infarct size after I/R injury, while the knockdown of miR-320 expression may have obviously protective effects on cells [22,50]. As previously mentioned, miR-320 seems to play a key role in myocardial I/R injury myocardial infarction, cell angiogenesis, cardiac hypertrophy, myocardial fibrosis and the protection of cardiac function.

In this study, the qRT-PCR results also indicated that the miR-320 expression levels in the I/R group were apparently up-regulated when compared with those in the sham group, suggesting that the miR-320 expression levels were closely associated with the myocardial I/R injury, and the increased miR-320 levels may have acted as a risk factor for myocardial I/R injury. miR-320 may play an important regulatory role in cell proliferation, cell differentiation and also cell apoptosis [51]. Furthermore, decreased endogenous miR-320 expression may reduce cardiomyocyte apoptosis when induced by simulated I/R, whereas over-expression of miR-320 increased sensitivity to I/R-triggered cell death [22]. Thus, both loss-of-function and gain-of-function studies have suggested that miR-320 expression is a negative regulator of cardio protection against I/R injury [24,52,53]. Our study further confirmed that the cardiac-specific expression of miR-320 has an increased expression in mouse models with myocardial I/R injury and a decreased miR-320 expression level, which may promote the improvement of heart function along with the treatment of antagomir-320.

However, several limitations in the present investigation still exist that merit our further exploration. Firstly, the trial size in the designed experimental rat model was relatively small, and the baseline characteristics shown in this paper were lacking regarding the adult male Wistar rats. Our study was focused on the animal model, which suggests that more experimental investigation among human populations is needed to contribute to the possibility of a target for the therapeutic intervention of myocardial I/R injury.

## 4. Materials and Methods

### 4.1. Animal Care

All rats were purchased from the Shanghai experimental animal center of the Academy of Sciences of China, and the procedures of this animal experiment were approved by the Animal Research Ethics Committee of the Second Hospital of Jilin University (JL2H20131220001; 20 December 2013). This study was conducted in accordance with the Helsinki Declaration of 1975, as revised in 1983.

### 4.2. Ischemia-Reperfusion (I/R) Injury Rat Model

The adult male Wistar rats weighing 240–280 g were anesthetized with 10% (0.4 mL/100 g) chloral hydrate by intraperitoneal injection before endotracheal intubation. Under aseptic conditions, the rats were placed in a supine position, and surface leads were placed subcutaneously. The electrocardiogram (ECG) monitoring was synchronously continued until the end of this experiment. After a left thoracotomy incision (1.5 cm from the third to fourth ribs), the left anterior descending coronary artery (LAD) was revealed by blunt dissection. A 6-0 nylon suture was placed around the left anterior descending coronary artery from the tip of the left auricle. Myocardial ischemia was performed by using a 6-0 nylon suture around LAD and tied for 60 min. Regional ischemia was confirmed by visual observation with the occluded distal myocardium of a pale color.

The 120 Wistar rats were randomly assigned into three groups: (1) the sham-operated group (sham group, *n* = 40); the rats underwent sham operation without coronary artery ligation; (2) the ischemia/reperfusion group (I/R group, *n* = 40), where the rats were treated with ischemia for 60 min; and (3) the I/R + antagomir-320 group (*n* = 40); 24 h before left coronary artery ligation, the rats were given an intramyocardial injection of antagomiR-320 (2 µg per day) at 1, 3, 5, 7 and 30 days after the operation.

### 4.3. Transthoracic Echocardiography

Echocardiogram indices of cardiac function and changes in hemodynamic parameters were recorded and analyzed using echocardiography at various time points (day 1, day 3, day 7, day 15 and day 30) after surgery. Transthoracic echocardiography was performed by using a high-resolution *in vivo* ultrasound imaging system with a 12-MHz transducer (hilipsSonos5500, Bio-Rad Corp., Hercules, CA, USA). When the picture on the screen was stabilized, the left ventricular ejection fraction (LVEF) and left ventricular fractional shortening (LVFS) were measured from the parasternal long-axis view at the mid-papillary muscle level with the accompanying software.

### 4.4. Hemodynamic Examination

Hemodynamic measurement was conducted immediately after echocardiography. The rats were transferred to a clean experimental platform and connected with a small animal ventilator (Medical equipment company, Shanghai, China). The right carotid artery was separated into proximal, distal and mid-regions. The proximal portion of the carotid artery was occluded with a bulldog clamp, and the distal portion was ligated. After routine disinfection and draping, this artery was punctured, and then, a micro-pressure sensing catheter (rinsed with heparin-saline) was placed into the artery. Subsequently, the clamp on the carotid artery was loosened, and the catheter was placed into the left ventricle. Pressure recording equipment was turned on; left ventricular end-diastolic pressure (LVEDP), left ventricular end systolic pressure (LVESP), maximum rate of left ventricular pressure rise (LVd*p*/d*t*_max_) and maximum rate of left ventricular pressure decline (LVd*p*/d*t*_min_) were recorded and measured by a BL-420F polygraph system (Taimeng Technology Co., Ltd., Chengdu, China).

### 4.5. Histological Examination

After the hemodynamic examination, a thoracic operation was performed with ventilator support and ECG monitoring; the heart of the rat was excised, and the blood supply area of left anterior descending (LAD) artery was dissociated. Myocardial tissue of the infarct zone was collected quickly. At the end of the experimental period, the rats were euthanized by cervical dislocation. Tissues samples of rats were cut into two parts: half of the samples were snap-frozen in liquid nitrogen until use. The remaining samples were fixed in 4% neutral-buffered paraformaldehyde for 24 h. Sections 5 μm in thickness were paraffin embedded according to the standard procedure. Then, the samples were stained with the hematoxylin and eosin (H&E). The degree of heart damage and photographs were obtained from each heart section (*n* = 3 sections per heart) under optical microscopy.

### 4.6. Measurement of Myocardial Fibrosis

The quantity of myocardial collagen fibrosis on sections stained with Sirius Red was measured by Image Pro Plus 6.0 (GelDoc2000, Bio-Rad). In each section, digital photographs were taken at five randomly selected fields. The myocardial fibrosis area percentage was calculated by the ratio of the stained area to the total area. The calculation of the collagen volume fraction is as follows: collagen volume fraction (CVF, %) = (total area of collagen/total area of image) × 100%.

### 4.7. Determination of Myocardial Apoptosis

Apoptosis of the heart sections was performed by terminal dUTP nick end-labeling (TUNEL) staining with an *in situ* cell death detection kit (Promega Co., Madison, WI, USA) in accordance with the instructions of the manufacturer. Briefly, the enzyme, TdT, was applied to incorporate digoxigenin-conjugated dUTP into the ends of DNA fragments. The signal of TUNEL was measured by an anti-fluorescein antibody conjugated with alkaline phosphatase. Graded alcohols were used to dehydrate the slides, which were then coverslipped with hematoxylin for counterstaining. For each slide, color video images of 280–360 μm fields were captured and digitized by use of a ×25 objective with a Sony DXC-760MD video camera, a RasterOps 24 XLTV video card and Media Grabber software on a Macintosh computer. The cells with clear nuclear labeling were defined as TUNEL-positive cells. The apoptotic cells were calculated as the percentage of TUNEL-positive cells using the following formula: number of TUNEL-positive cell nuclei/(number of TUNEL-positive cell nuclei + the number of total cell nuclei) × 100%.

### 4.8. MicroRNA Extraction and qRT-PCR

We isolated the total RNA by using miRNeasy^®^ mini Kit RNA (QIAGEN, Valencia, CA, USA), in accordance with the manufacturer’s protocol. The cDNA was synthesized by using the TaqMan^®^Reverse Transcriptase Kit (ABI, Forest City, CA, USA) with 3 μL of total RNA from each sample. The quantitative real-time RT-PCR was performed using the Maxima^®^SYBR Green/ROX qPCR Master Mix (2×) (Fermentas, Burlington, ON, Canada) in an ABI 7500 Real Time PCR System. The primers used for miR-320 in the present study were as follows: forward primer: 5'-AAAAGCTGGGTTGAGAGGG-3'; RTprimer: 5'-GTCGTATCCAGTGCGTGTCGTGG-AGTCGGCAATTGCACTGGATACGACTTCGCCCT-3'; reverse primer: 5'-TGCGTGTCGTGGAGTC-3'. All reactions were incubated at 95 °C for 10 min, followed by 40 cycles for 15 s at 95 °C and at 60 °C for 1 min. The U6 served as an internal control. Data for miR-320 copies were determined as the relative quantification, which was measured by using the 2^−ΔΔ*C*t^ method.

### 4.9. Statistical Analysis

All data analyses were performed using SPSS 18.0 software (SPSS, Inc., Chicago, IL, USA). Data were expressed as the mean ± standard deviation (mean ± SD), frequencies or the median with interquartile ranges (IQR). The χ^2^ test was used to compare percentages. The Student’s *t*-test and one-way analysis of variance (ANOVA) were used for normal distribution variables. Non-normally distributed variables were calculated by the Mann–Whitney’s *U*-test. The Fisher exact test was used to compare nominal variables between the two groups. A probability value of less than 0.05 was considered as statistically significant.

## 5. Conclusions

To summarize, our study results support the view that the expression of miR-320 might be up-regulated in the rat model of myocardial I/R injury, and miR-320 might contribute to the prevention of left ventricular remodeling after myocardial I/R injury. Consequently, decreased miR-320 expression may, in turn, be considered as a possible predictive factor in reducing the risk of left ventricular remodeling risk after myocardial I/R injury and improving cardiac function.
